# Obstacles in access to care for children

**DOI:** 10.1186/1824-7288-41-S2-A66

**Published:** 2015-09-30

**Authors:** Nathalie Simonnot, Pierre Chauvin

**Affiliations:** 1 Doctors of the World – Médecins du Monde (MdM) International Network, Paris, 75018, France; 2 Sorbonne Universités, UPMC Univ Paris 06, INSERM, Pierre Louis Institute of Epidemiology and Public Health (IPLESP UMRS 1136), Paris, 75012, France

## Background

Doctors of the World – Médecins du Monde (MdM) provides free frontline medical and social services for people facing barriers to the mainstream healthcare system. Child poverty rate has been soaring since 2008, especially in Spain and Greece. Restrictive laws across Europe often hinder any access to preventive and curative care, including for children whose parents are undocumented migrant (UDM) or lack health coverage. For example, in Germany, civil servants have to denounce children of UDM. In Spain, the legal restrictions on UDM negatively impacts their children rights. In the UK, if one of their parents is not registered at a GP, the children will not be accepted. In Greece, in 2014, around a third of the population has no more health coverage.

## Materials and methods

Data collected by MdM teams with 23,040 people in 25 cities in 10 European countries with the use of common social and medical questionnaires administered during face to face social and medical consultations [1].

## Results

Most of the 623 children for whom we have detailed data live in harsh conditions (slums, overcrowded and health-threatening housing, or even out in the streets). Less than half were immunised against tetanus (42.5%), pertussis (39.8%), hepatitis B (38.7%) and measles, mumps and rubella (34.5%). Also, 38.8% of the parents didn't know where to go to get vaccination for their children. Children of UDM also fear their parents’ arrest, have to move from place to place hindering continuity in care and a stable social life. Migrants’ children are put at risk because of the barriers to antenatal care faced by their mothers. Even when children have theorically full access to care, administrative barriers too often stop their access.

## Conclusions

European States must offer universal public health systems built on solidarity, equality and equity (and not on profit rationale), open to everyone living in Europe. They should ensure that all children residing in Europe have full access to immunisation programs and paediatric care. All pregnant women must have access to abortion, antenatal and postnatal care, and safe delivery. Launched in April 2014, the Granada Declaration [2] calls for a better protection of migrants’ health and healthcare, including that of UDM. In accordance with the World Medical Association's Declaration on the Rights of the Patient, we urge all health professionals to provide appropriate medical care to all without discrimination, and to refuse restrictive legal measures that alter medical ethics.
